# What Young People Who Have Experienced Maltreatment Want From Out-of-School Sex and Relationship Education: A Qualitative Synthesis of Participatory Research With Young People

**DOI:** 10.1177/15248380251349786

**Published:** 2025-11-29

**Authors:** Tim Moore, Jodi Death, Sebastian Trew, Gina Steer, Jessica Dickson

**Affiliations:** 1 Australian Catholic University, Fitzroy, VIC, Australia; 2 Queensland University of Technology, Brisbane, Australia; 3 Royal Melbourne Institute of Technology, VIC, Australia

**Keywords:** sexual assault, violence exposure, prevention, prevention of child abuse, child abuse

## Abstract

Childhood maltreatment can have profound and lasting impacts on survivors, often necessitating a raft of therapeutic and trauma-informed services. In recent studies, young survivors have highlighted the potential for sexual and relationship education (SRE) to provide young people opportunities to make sense of and seek support related to their maltreatment but have reflected that their experiences of SRE are rarely responsive to their needs. Given that many are not able to access SRE at school, they have argued for targeted programmes to be tailored to their needs. This systematic review explores what young people who have experienced maltreatment want and need from SRE in non-school settings. Drawing on seven research papers published between 2000 and 2023, where young survivors were engaged directly in qualitative studies, the paper identifies key considerations for creating trauma and survivor-informed SRE. Findings emphasise the importance of universal SRE programmes in acknowledging the link between childhood maltreatment and risk behaviours, accommodating survivors’ unique experiences and educational needs in SRE design, and addressing their heightened vulnerability to future harm. It advocates for more targeted and intensive programmes that are accessible to young survivors at key points while linking them up to services to help them reclaim their sexualities. Moreover, the study advocates for a nuanced understanding of young survivors’ resilience and the need for sensitivity when facilitating trauma-informed programmes. It stresses the value of working with survivors to shape SRE and the ways that they would like to play a role as peer educators and advocates.

## Background

During adolescence, young people undergo a series of physical, psychological, and neurological changes during a time marked by transition, growth, and exploration. Despite adolescence often being characterised in terms of stress and storm and adolescent sexuality described in negative terms (Fava & Bay-Cheng, 2013), young people discover new ways of relating, of experiencing love and positive relationships outside of their families and of exploring new talents and a greater sense of independence (Tolman & McClelland, 2011).

Despite being exposed to more sexual content and being able to access more information about sex and relationships now more than in any previous generation (Simon & Daneback, 2013), young people continue to desire opportunities to learn, to be able to critically appraise the information that they receive and to speak about ways that they might better manage relationships, sex and sexuality (Astle et al., 2021; Park & Kwon, 2018).

Research also demonstrates that by late adolescence, almost 40% of young people (particularly young women) have also been exposed to sexual harassment, abuse, and violence – perpetrated both by adults and peers (Haslam et al., 2023; Vizard et al., 2022).

Childhood maltreatment can have profound and enduring impacts on young people’s physical health, mental health, psychosocial adjustment, on their relationships, engagement with school and their sense of identity (Angelakis et al., 2020; Carr et al., 2020). Research has found that some young victim-survivors may go on to experience homelessness, mental health challenges, early pregnancy, demonstrate their own harmful sexual behaviours (HSBs) and interact with the child protection and youth justice system (Angelakis et al., 2020; Graf et al., 2021; Liu et al., 2021; Malvaso et al., 2020). Young people who have experienced childhood maltreatment and these further challenges are also at greater risk of experiencing subsequent abuse during their adolescence and throughout their lives (White et al., 2015).

### The Need for Quality Sexual and Relationship Education That Meets the Needs of Young Survivors

As there is a strong desire from young people generally to learn about respectful, healthy, and safe relationships, advocates have argued that young victim-survivors be provided with appropriate, responsive and trauma-informed sex and relationship education (SRE) that better meets their needs (Fava & Bay-Cheng, 2013).

SRE has been defined as “any combination of learning experiences aimed at facilitating voluntary behaviour conducive to sexual health” (Lameiras-Fernández et al., 2021). According to UNESCO, “it aims to equip children and young people with knowledge, skills, attitudes, and values that will empower them to realise their health, well-being and dignity; develop respectful social and sexual relationships; consider how their choices affect their own well-being and that of others; and understand and ensure the protection of their rights through their lives” (Joint United Nations Programme on HIV/AIDS, 2021, p. 12).

Respectful Relationship Education is one form of SRE that aims to improve young people’s skills “to build and sustain future strong and stable relationships” (Janssens et al., 2020) to reduce intimate personal violence during adolescence and in adulthood (Benham-Clarke et al., 2023). Such SRE has the potential to provide young people with opportunities to better understand their experiences, to manage their shame and self-blame and distorted views of sexuality (if they hold them) and to “account for the positive potential of sexuality in the lives of young people with trauma and maltreatment histories” (Fava & Bay-Cheng, 2013).

However, until recently, SRE programmes have rarely taken into account the needs of survivors or those young people who are currently in unsafe relationships (Fava & Bay-Cheng, 2013) nor considered the ways that universal programmes can be more attuned to the impacts of their trauma or be provided in such a way that enables young people to develop healthy sexual relationships and behaviours. Instead, advocates have argued that they may, unwittingly, perpetuate “the idea that a history of victimization means foreclosing on future desire, pleasure and fulfillment” (Fava & Bay-Cheng, 2013). Programmes that are not aware of, responsive to or keep the needs of young survivors in mind can be unhelpful, harmful, or even triggering. In Australian studies, young people have expressed a wish for SRE to be reoriented, and survivors have sought opportunities to inform what and how SRE is provided (see Moore & McArthur, 2022; Moore & McDougall, 2026).

## Research Aim and Method

The aim of our review is to identify the key characteristics of SRE that are valued by young victim-survivors and those who have otherwise experienced maltreatment to inform the development of survivor and trauma-informed programmes. In this paper, we synthesise findings from participatory studies that consider *what, where, how, and by whom* young people want and need SRE to be provided in non-school settings. The paper complements other syntheses conducted with young people in schools (Pound et al., 2016).

Three research questions guided our review, including: (a) What content do young survivors value in SRE in non-school settings? (b) How do young people want SRE to be delivered: including when, where, how and by what means? and (c) What attributes do young people value in those facilitating SRE?

This review was conducted as part of a study funded by the the National Centre for Action on Child Sexual Abuse and conducted by researchers from the Australian Catholic University and the Queensland University of Technology. The broader project aims to engage young people, including those who have experienced maltreatment, and other educators, practitioners, and academics to co-design a set of trauma and survivor-informed principles and indicators of quality to help shape future SRE programmes.

### Search Strategy

The review commenced with a scoping search to identify previously published literature or systematic reviews using broad search parameters. The Database of Abstracts of Reviews of Effects and the Cochrane Library were searched, and any relevant registered protocols were also reviewed in Prospero. Search terms underwent numerous refinement processes as a result of scoping searches, consultation with co-authors, and consultation with a librarian. As we were particularly interested in the views of young people who had experienced maltreatment, we developed a search strategy that included permutations of the terms: survivor, victim, child sexual abuse, sex, relationship, health, education, and variations of keywords such as SRE. Keyword searches were restricted to title or abstract only. We then applied subject headings in each database and combined using OR with the relevant keyword search string. An example search strategy used in the Cumulated Index in Nursing and Allied Health Literature. We then conducted additional searches to identify studies that combined sex and relationship education (SRE) with young survivors and evaluations of interventions that engage this group. The searches were developed based on those utilised in previous syntheses of qualitative studies conducted in school-based settings (Pound et al., 2016). We then utilised limiters so that only peer-reviewed, empirical studies that were qualitative and participatory in nature published in the English language were included. Studies were considered “participatory” when they directly involved young people rather than relying on proxies such as parents, teachers or others.

Five databases were searched from January 2000 to November 2023, including: Campbell Collaboration, CINAHL, Medline Complete, APA PsycINFO and SocINDEX (Ebsco).

### Inclusion/Exclusion Criteria

In Australia, Sexual Health and Relationship Education includes formal and informal programmes with content related to sex and sexuality, sexual health, intimate violence prevention and respectful relationships. During their primary school years (e.g. when aged between 5 and 12), Australian children are provided child abuse prevention education, most often “Protective Behaviours” programmes, which teach them skills in identifying and raising concerns about the behaviours of others. Given how these programmes are different to SRE in regard to the age at which they are provided, the content that is delivered and the outcomes that are sought – such studies were excluded from this meta-synthesis.

For this meta-synthesis, researchers decided to include studies if the title and abstract indicate that they met the following criteria: (a) Published between January 2000 and November 2023; (b) Available in English; (c) Reported on qualitative participatory studies that directly engaged young people between the ages of 12 and 18 or sought their views on supports provided to them at this age; and (d) Reported on their experiences of sexual health and/or relationship education.

After retrieval, duplicates were removed using Covidence before each title and abstract was screened for eligibility. Each article was then read, in full, to ensure that they met the eligibility criteria. Subsequently, an additional eligibility criterion was added, namely that the study’s sample targeted or included young people who had experienced maltreatment and/or were victim survivors.

**Figure 1.**
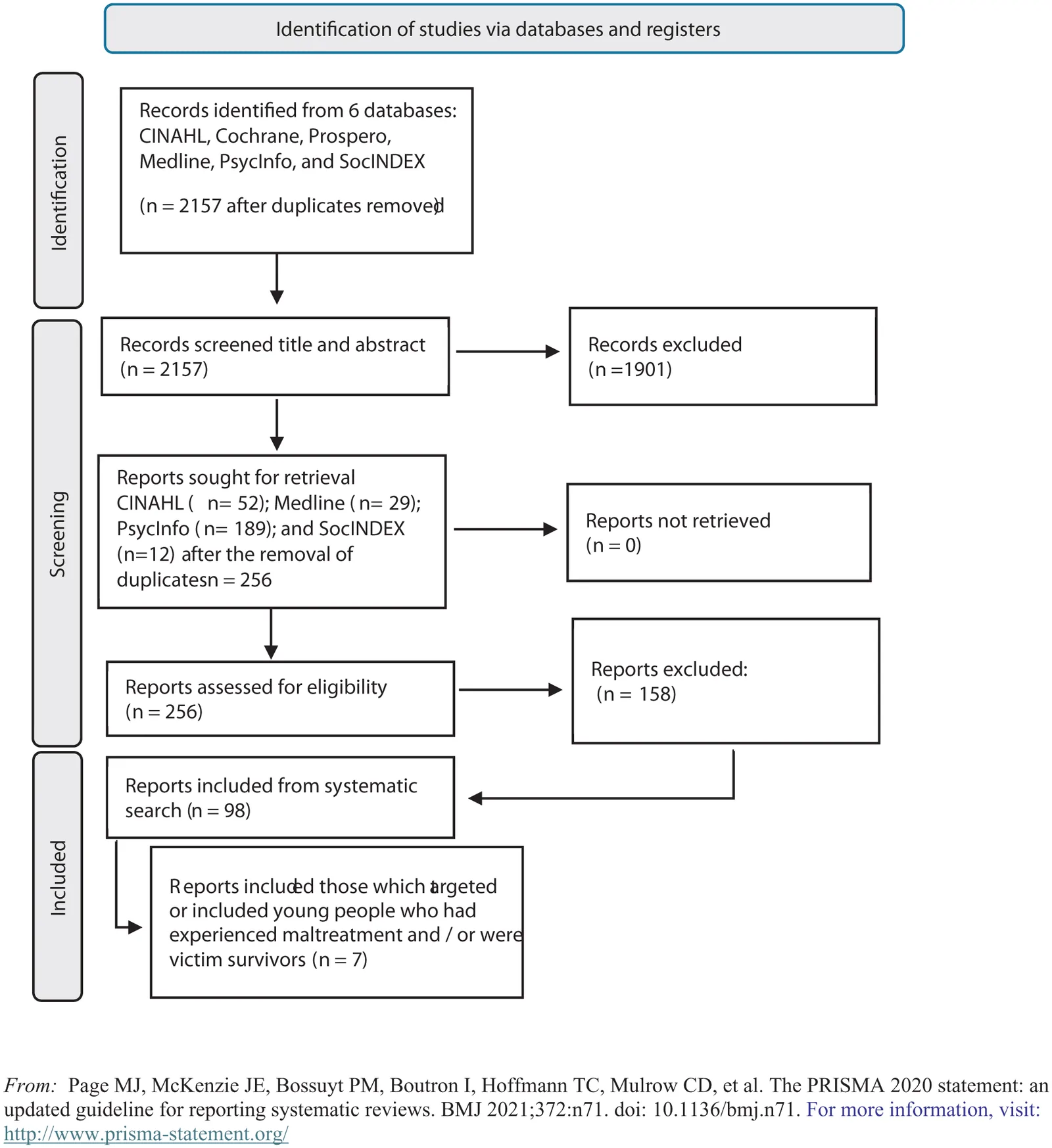
PRISMA flowchart. *Source*: Page et al. (2021). For more information, visit: http://www.prisma-statement.org/.

Studies were excluded if the research: (a) Was published as a thesis or report; (b) Was conducted in a country where young people’s experiences of SRE may not be comparable to those of young people in Australia (e.g. studies from Africa, South America were excluded while studies from the North America and Europe, e.g. were included); (c) Were not “participatory” as they did not directly engage young people (e.g. used adult proxies to reflect on young people’s experiences, utilised secondary data or were other syntheses); and (d) Did not include or consider the evidence provided by young participants in the studies. Research studies where young people were engaged alongside their parents, carers or workers were included. The identification and screening process is presented in Figure 1.

#### Critical Appraisal

A critical appraisal is a vital first step in conducting a meta-synthesis (Ludvigsen et al., 2016). We used the Critical Appraisal Skills Program tool to assess the quality of the research presented. Given the nature of the studies included, we also assessed papers on their “youth-centredness” utilising a set of questions developed in a previous analysis (Noble-Carr et al., 2021). This set of additional questions considers the extent to which young people helped design, develop, implement, and inform analysis, findings, and dissemination. Tim Moore and Jodi Death independently assessed each study against the relevant study design checklist. Studies were rated against 10 appraisal criteria, depending on the checklist used with rate response options for each criterion recorded as *yes, no*, or *unclear*. A final score of between 0 (*indicating low quality*) and 1 (*indicating high quality*) was agreed only when two assessors rated 100% agreement on each item. Sebastian Trew adjudicated on discrepancies.

As is recommended, no articles were excluded based on this assessment, but the nature and extent to which young people have played a part in shaping SRE research will be discussed elsewhere.

Our quality assessment, including our appraisal of youth-centredness, is included in Table 1.

**Table 1.** Quality Appraisal and Youth Centredness Assessment.

Domain	Aparicio et al. (2021)	Aparicio, Kachingwe et al (2019)	Aparicio, Shpiegel et al. (2019)	Lindroth (2014)	McKibbin et al. (2017)	Opara et al. (2022)	Rew et al. (2002)
Quality Appraisal							
Clear statement of aims	1	1	1	1	1	1	1
Qualitative method is appropriate	1	1	1	1	1	1	1
Design appropriate to answer questions	1	1	1	1	1	1	1
Recruitment strategy appropriate	1	1	1	1	1	1	1
Data collected addresses issue	1	1	1	1	1	1	1
Positionality is described	0	0	0	0	1	0	0
Ethical issues addressed	1	1	1	1	1	1	1
Analysis is rigorous	1	1	1	1	1	1	1
Statement of findings clear	1	1	1	1	1	1	1
Value of research is demonstrated	1	1	1	1	1	1	1
Youth engagement							
Governance	0	0	0	0	0	1	0
Study design	0	0	0	0	0	1	0
Co-researchers	0	0	0	0	0	1	0
Member-checking	0	0	0	0	0	1	0
Analysis and interpretation	0	0	0	0	0	1	0
Dissemination	0	0	0	0	0	0	0

#### Data Extraction and Analysis

Data were extracted and then analysed with the aid of the computer software program, Non-Versioned Information Versatile Outcomes (NVIVO), in which the data were stored, sorted, and coded (Bazeley, 2021). Data were coded deductively into themes, framed by the three research questions, and then inductively to more fully understand how the theme emerged in different contexts and with different cohorts of young people (Padgett, 2016).

In the following section, we report on the findings from this meta-synthesis. To enhance this papers’ credibility (Finfgeld, 2003) and to be true to our youth-centred approach, we include direct quotes from young people so that their voices are prioritised but also make reference to observations made by adults (e.g. parents, carers, and workers) to provide context to their views.

## Findings

### Nature of Included Studies

To better understand the nature and scope of existing studies, we extracted information on each regarding the focus of the study, the country within which data were collected, the types of maltreatment of participants reported, the setting within which the study was completed (where relevant), the gender and age range of participants and the methodology and methods that were used to collect data.

Seven papers, drawn from the broader sample (of 98 papers), that reported on empirical, qualitative studies published between 2002 and 2023 and engaged young victim-survivors met our inclusion criteria. Five of the studies were conducted in the United States, one in Sweden and one in Australia. These studies reported on the experiences of young people who were engaged with the child protection systems (including group homes [*n* = 2], homeless services [*n* = 2], and young people who had left care [*n* = 2]) or were receiving treatment for HSBs [*n* = 1]). Many of the papers were exploratory, while two (Aparicio, Kachingwe, et al., 2019; Lindroth, 2014) reported young people’s reflections on their engagement in existing programmes and how it was delivered. Across the samples, almost all participants reported that they had experienced some form of maltreatment, including physical, emotional, sexual abuse, and/or exposure to family violence or neglect. Included research projects sometimes focused primarily on sexual health education (*n* = 3), with the remaining focusing on both sexual health and relationships.

132 young people participated in the included studies, with the number of young people in each sample ranging from 5 to 51. The age ranges of young people in studies ranged from 14 to 22 years. Three of the studies only included female participants, and less one-third of participants across studies were male. Two studies reported that they included transgender young people. Many of the young people were from culturally or linguistically diverse communities. Recognising the sensitivity of the topics being discussed with young people, two studies used interviews to elicit data, four engaged young people in focus groups and one included a mix of both methods. An overview of included studies is included in Table 2.

**Table 2.** Overview of Completed Studies.

Article	Country	Maltreatment types	Sample	Age range	Sex education/respectful relationships/mix	Methods
Aparicio et al. (2021)	United States	Child abuse (broad)	*N* = 15. Including 6 young women; 14 culturally or linguistically diverse young people	16–20 years	Mixed	Focus groups
Aparicio, Kachingwe et al. (2019)	United States	Child abuse (including physical and sexual abuse; neglect; exposure to Intimate Partner Violence)	*N* = 51. Including 51 young women	14–22 years	Sex education	Interviews and focus groups
Aparicio, Shpiegel et al. (2019)	United States	Child abuse (including neglect, physical and sexual abuse, and IPV)	*N* = 6	19–22 years	Reference to support needs broadly	Interviews X3pp
Lindroth (2014)	Sweden		*N* = 14 (including 7 young women)	14–19 years	Mixed (sex education + Respectful Relationship Education)	Focus groups
McKibbin et al. (2017)	Australia	Child abuse (including IPV, sexual and physical abuse)	*N* = 14	16–21 years	Sex education	Interviews
Opara et al. (2022)	United States	Child protection involvement	*N* = 10	17–20 years	Sex education	Focus groups
Rew et al. (2002)	United States	Homelessness (including with histories of maltreatment)	*N* = 22	16–20 years	Sex education	Focus groups

### Understanding the Lives of Participants Including the Intersectionality of Maltreatment and Other Challenges

Across the studies, almost all (>95%) participants reported that they had experienced some form of maltreatment: including physical, sexual, and emotional abuse, exposure to family violence, neglect, or other forms of harm (see Table 1 for more detail). In many instances, authors made links between these early experiences and a range of other challenges, including engagement with the child protection system, early school leaving, homelessness, the selling of sex, criminality, engaging in risky sexual behaviour, using HSB and problematic alcohol, or other drug use. As one young person recalled:

If I didn’t experience family violence, I wouldn’t have been introduced to the crimes and stuff, because whenever I ran away from home, I went and met up – I met bad people. (Young person, male, 16) (McKibbin et al., 2017, p. 216)

Although most authors did not aim to demonstrate causality, participants in the studies often made links between their maltreatment and its impacts and the contemporary challenges that they face. For example, in McKibbin’s et al. (2017) study with young people using HSBs, participants reported that their own exposure to maltreatment caused family conflicts that led to prolonged periods away from home and exposure to criminal activity, while others reported that it caused them to have skewed views about sexual activity (and when it was appropriate and not), and unresolved anger and distress all arising from their maltreatment which influenced their own HSB.

As experiences of childhood maltreatment influenced the challenges that young people faced in their adolescence, so did the settings within which they now lived influence their experiences of sex, relationships, and SRE. For example, many young people reported that due to their care experience or homelessness they were no longer engaged in school and had little or no access to formal sexual health or relationship education (Aparicio, Shpiegel, et al., 2019). Other young people (Opara et al., 2022) observed that as they were now estranged from their families, they were now unable to engage in discussions related to sex and relationships. Being in foster care, group homes or homelessness services meant they were restricted in what they could talk to workers and peers about (Lindroth, 2014; Opara et al., 2022). As one participant observed:

I really never had my own parents to tell me about sex. I have been in like seven different foster homes and sex was the last thing on their minds. I was just trying to survive, and they were trying to figure out whether they wanted me. (Opara et al., 2022, p. 5)

As a result, young people often reported that they had to find information about sex, sexuality, sexual health and relationships on their own: “*I learned about sex from pamphlets I picked up or from television, no one talked to me about it*” (Opara et al., 2022, p. 5). They did, however, express an interest in receiving support from families and the services with which they interact (Aparicio et al., 2021; McKibbin et al., 2017; Opara et al., 2022).

### What Content Do Young Survivors Value in SRE in Non-School Settings?

Across the seven studies, young people identified a whole range of content areas that they either had appreciated exploring within existing programmes or reflected that they would have benefited from learning if they had been given the chance.

#### Abuse, Inappropriate Sexual Relationships, and Rape

As noted above, young people both accounted for their childhood experiences of sexual abuse, talked about inappropriate adult–child relationships and their exposure to inappropriate “adult knowledge” and their ongoing exposure to unhealthy relationships and sexual encounters (Aparicio, Kachingwe, et al., 2019; Aparicio, Shpiegel, et al., 2019; Aparicio et al., 2021; Lindroth, 2014; McKibbin et al., 2017). They also reflected on how their childhood experiences can influence young people’s current exposure and ability to exit unsafe relationships. As Aparicio et al. (2021, p. 114) reported:

Youth also described how unhealthy relationships could be especially difficult to manage for youth in foster care . . . “because obviously people in care are in care for a reason . . . adding on unhealthy relationships to those other reasons does not make a good mix.” . . . [and] As one youth explained, “sometimes they don’t know that they’re actually in that, like, that situation. They don’t even know it”, and another stated, . . . “I don’t think a lot of people know how to get out of them.”

However, in some papers (Aparicio et al., 2021; Opara et al., 2022), young people and adults reported that they believed that young people were not always adept at appreciating the interplay between their pasts and their current relationships and challenges. As one social worker noted in Aparicio et al.’s (2021, p. 1144) paper:

I think that [youth in care] may not fully understand how their past trauma is affecting their mental health and their views of sex and sexual activity, or why they do the things they do. So, I think it would be good if we could maybe delve more into trauma and behaviors as a result.

As a result, there was a view that SRE needed to include opportunities to build young people’s understanding and insight to ensure that they could utilise what they had learned in these educational programmes. Without these opportunities, some young people (and authors) argued that they were ill-equipped to have “sex without the appropriate knowledge to engage in safe and pleasurable sex” (Opara et al., 2022, p. 5).

Young people advocated the need for SRE to include details on the raft of risks to young people’s safety to enable young people to make sense of their past abuse, to better appreciate that such relationships were problematic and to prevent them from engaging in unsafe or unhealthy relationships within which they might perpetrate or be exposed to sexual assault or rape. Although authors recognised that some universal or school-based SRE content includes details about unhealthy relationships, the types of concerns raised by young people in the included studies were significant and pressing and required discussions to focus on risks more explicitly. For example, young people in care talking about sexual assault and rape within relationships observed in Opara et al.’s (2022, p. 5) study: “that traditional ways of teaching negotiation skills through sexual health education was too simple. ‘These aren’t real-life scenarios, these scenarios are too clean,’ said Sasha.”

#### Sexual Health

For the purposes of this paper, we define sexual health broadly and not only as “the absence of disease, dysfunction or infirmity” but also “having pleasurable and safe sexual experiences, free of coercion and violence” (WHO, 2006). In most of the papers, young people reported that they and young people like themselves would like education programmes to explore issues of sexual health. They felt that they would benefit from learning about basic physiology and anatomy (including puberty and changes in their bodies), about reproduction and pregnancy, sexual health (including how to prevent sexually transmitted infections, particularly HIV) and contraception (including condom use). In regards to understanding “how to have sex,” young people in Rew’s et al. (2002) study, talked about the need to understand sex with both partners of the same and different genders and the risks associated with having sex with strangers or for money.

Although these topics appeared to be somewhat like what is currently provided in universal (and often school-based) SRE programmes, young people provided detail about their needs for such information and education to be tailored to their experiences. For example, in Lindroth’s (2014) study, which included young people who were “sex-selling,” young people reported a lack of awareness about the illegality of sex work and of adults having sex with young people. They talked about the challenges of negotiating condom use when adults were paying them to have sex or, in Rew’s et al. (2002) study, when young people were having “survival sex” (e.g. trading sexual activity for money, drugs, or shelter). Other young people argued that, as a result of their different experiences, SRE needed to be tailored to their experiences and be responsive to their needs (Aparicio et al., 2021).

These young people also highlighted the need for sex education to focus on the interplay between sex, consent and alcohol or other drug use and how to use and engage in sexual activity safely (Aparicio et al., 2021; Lindroth, 2014; Opara et al., 2022; Rew et al., 2002). Participants reported that while affected by alcohol, they were less inclined to use contraceptives, found it more difficult to negotiate consent (and condom use) and to keep themselves safe (Aparicio et al., 2021; Lindroth, 2014). Sessions focusing on ways to deal with these issues were seen as pressing by authors who recognised the high incidence of problematic alcohol or other drug use both among survivors of maltreatment and those engaged in the care and homelessness systems (Aparicio et al., 2021; Rew et al., 2002).

“you shouldn’t be drinking that much alcohol,” and if you are “too fucked up . . . to ask a person or not about their sexual past . . . then you probably aren’t going to be very good at having sex.” (Rew et al., 2002, p. 171).

#### Gender Roles and Expectations

Young people in a number of studies were engaged in discussions about gender norms, heteronormativity, and different sexualities (Lindroth, 2014; Opara et al., 2022). Although young people did not always identify their need or desire to discuss these issues, authors reflected that a number of concerning attitudes related to gender, sexuality, and relationships could be observed, particularly within foster care and homelessness contexts (Lindroth, 2014; Opara et al., 2022). Young people and authors reflected that, often due to their past exposure to intimate partner and family and domestic violence, some young people had developed notions of masculinity and femininity that were problematic and may sustain them in unhealthy relationships (McKibbin et al., 2017).

In her study, McKibbin et al. (2017) also made connections between pornography and young people’s HSBs (although only three participants made the connection themselves) while young people in the Lindroth (2014) paper appeared to normalise sexualised images and did not appreciate how other young people or staff might feel about the way that women and sex were conveyed within them.

Issues of male entitlement to sex within relationships, young women’s expectations related to pregnancy and parenting and young men not taking responsibility for contraceptive use are examples highlighted in the literature that might be addressed in SRE (Aparicio, Kachingwe, et al., 2019; Aparicio, Shpiegel, et al., 2019; Aparicio et al., 2021; Lindroth, 2014; Opara et al., 2022). Empowering young people to adopt positive notions of masculinity and femininity and being open to conversations about diverse sexualities were seen as important aspects of SRE (Aparicio, Kachingwe, et al., 2019; Aparicio, Shpiegel, et al., 2019; Aparicio et al., 2021; Lindroth, 2014; Opara et al., 2022).

#### Sexual Relationships

Across five groups, young people also valued or expressed the need for SRE to include discussions on how to manage sexual relationships and for these discussions to be relatable to young people and be responsive to their needs. For example, in three studies, young people recognised that due to their past traumas, they were often quick to engage in relationships (including those that were sexual in nature), were often unskilled in identifying risks and determining whether interactions with their partners were healthy or not.

Again, there were nuances for different groups of young people. For example, young people in Aparicio’s studies living in care (Aparicio, Kachingwe, et al., 2019; Aparicio et al., 2021) talked about the fact that they and their peers were more at-risk of engaging in risky or unhealthy sexual relationships (or to become pregnant), and saw relationships as the best way to receive the affection and love that they were not otherwise receiving and saw sex as a way of confirming their relationships (Aparicio, Kachingwe, et al., 2019; Aparicio et al., 2021).

Young people who had engaged in HSBs believed, for example, that it was important for them to be taught more about what is appropriate and what is not (McKibbin et al., 2017). They advocated for discussions to go beyond consent and explore the morality of engaging younger children in sexual activity. Others believed that if they had received appropriate and responsive SRE that such behaviours might not have occurred.

### How Do Young People Want SRE to Be Delivered?

Before presenting findings in relation to young people’s preferences in terms of delivery, it is important to note that some young people reported that they either had no access to SRE or found it unsatisfactory (Aparicio, Kachingwe, et al., 2019; Opara et al., 2022). In three studies, participants reported that due to their poor engagement in schools they had not attended school-based programmes. Other young people reflected that the universal programmes (mostly provided in schools) varied greatly and were often of low quality. As Opara et al. (2022, p. 4) summarise: *During these focus groups, it was evident that youth were critical of their families, the child welfare system, and schools in providing inadequate access to sexual health education.* In the following sections, we discuss what young people want from SRE in non-school settings. Although young people did not advocate against hosting SRE programmes in schools, they were much more likely to speak positively about programmes offered in community-based settings while providing guidance on how such education might be best delivered.

#### When SRE is Provided

In four of the seven studies, young people (and researchers and professionals supporting youth) reported that there were pivotal points at which they wanted and needed access to good age-appropriate information and education about sex, sexuality, and relationships.

Firstly, there was a view that SRE needed to be provided early – preferably prior to puberty (Lindroth, 2014; McKibbin et al., 2017; Opara et al., 2022). For young people in or leaving care, for example, early SRE was seen as a way of dispelling false assumptions about sex, sexuality and pregnancy while empowering young women, in particular, to have expectations about what they might want in a relationship while identifying other ways to have their needs met (e.g. for love, connection, and positive identity) (Aparicio, Kachingwe, et al., 2019; Aparicio, Shpiegel, et al., 2019; Aparicio et al., 2021). Young people believed that they needed to have received this information and education a long time prior to their first sexual encounters and relationships.

Similarly, young people in McKibbin’s et al. (2017) study reflected that they would have liked to have better understood sex and sexual relationships (particularly what was appropriate and not) before they hit puberty to prevent their engagement in HSB.

For me, it was honestly teachers who told me about sex and that was when I was older and already had sex so it seemed too late. (Opara et al., 2022, p. 5)

In addition, to calls for SRE to be available to young people early in life, several authors pointed to other points at which SRE was vital. Young people in Aparacio et al.’s studies, for example, felt that to empower young women and to reduce the likelihood of early pregnancy and parenting, SRE was required when young people hit puberty, when they started engaging in sexual relationships and when they fell pregnant (Aparicio, Kachingwe, et al., 2019; Aparicio, Shpiegel, et al., 2019; Aparicio et al., 2021). The type of SRE needed at these points varied from information about healthy relationships and sex, contraceptives and pregnancy provided from puberty onwards to more targeted information and education about reproductive options when young women fell pregnant (Aparicio, Kachingwe, et al., 2019; Aparicio, Shpiegel, et al., 2019; Aparicio et al., 2021).

As a means to reduce the likelihood that young people might utilise HSBs, young people in McKibbin’s et al. (2017) study reported wishing that SRE had been provided: before or during puberty; and after they themselves had experienced maltreatment (to better understand their experiences and for their skewed views on sex, relationships, and abuse to be corrected):

Before [puberty] occurs or definitely, what’s been brought up is . . . get the school and the parents into sex education and do it before [puberty] occurs. (Young person, male, 17) (2017, p. 215).

### From Whom and Where Do Young People Want to Receive SRE?

Young people provided feedback on and identified those from who they believed they would feel most comfortable receiving SRE. Primarily, where possible, they valued opportunities to receive information, support and guidance from their parents or families but also spoke about the value of health and community services. Young people in the included studies often spoke about the existing programmes (including at school, in youth services and community settings) as being unhelpful and unresponsive to their needs.

#### Parents and Family

When asked about who they could turn for sex and relationship advice, many talked about their families and parents (Aparicio et al., 2021; McKibbin et al., 2017; Opara et al., 2022) – even when they were not living with them (Aparicio et al., 2021). However, some reported that “*some people don’t have the right relationship to talk about shit like that [sex] with their family*” (Opara et al., 2022).

Even when young people felt that parents and family might be a good source of information and education about sex and relationships, they conceded that often parents did not feel comfortable having these conversations and were not always equipped to answer their children’s questions (Aparicio et al., 2021; McKibbin et al., 2017): “*I wish that parents were more open to talking to their kids. Like even at like once, the kid starts puberty, I feel that they should be talking about sex*” (Opara et al., 2022, p. 4).

Similarly, participants felt that for young people to be comfortable in *receiving* information, their parents (or caregivers) needed to be proactive and to “*get a bond. Create a bond. Get a strong ‘I got you’ [understanding with your kids*]” (Opara et al., 2022, p. 1142).

#### Foster Carers, Youth Workers and Other Professionals

Young people spoke about professionals to whom they could go to for support, including school counsellors, group home staff, foster carers, and professionals, case workers and other supportive adults (Aparicio, Kachingwe, et al., 2019; Aparicio et al., 2021). Young people stressed the importance of trust, recognising that some were adult-wary, “due to prior experience with caregivers and others in their lives.” This, they believed, “requires serious and long-term commitment” (Aparicio et al., 2021, p. 1142).

Like concerns about parents’ willingness and ability to meet their children’s educational needs, young people felt that professionals were not always able to provide advice on sex and relationships and that most “[adults] avoid the topic of sex [and] Curiosity kills the cat” (Opara et al., 2022, p. 4).

Given that some professionals working with young people may not have the expertise to provide them adequate and responsive SRE, there was a call for programmes to be concurrently provided: that parents, foster carers, youth workers and others might benefit from education and support while young people were engaged in programmes (McKibbin et al., 2017; Opara et al., 2022).

#### Health Services

Young people appeared to have varied views about health services as a source of information and education. For example, in the Wahine Talk project (Aparicio et al., 2021), which aimed to improve young people’s access to sexual health education, the team observed that many of the young people were reluctant to attend health centres, particularly if they were unaccompanied, reporting that the space was not youth-friendly and staff, inaccessible. Providing young people support to feel comfortable in spaces, to be reassured about what might happen there and for staff to be welcoming was seen as important (Lindroth, 2014).

### What Attributes Do Young People Value in SRE Programmes and Those Facilitating Them?

Much of the data collected from young people about what young people want and need from SRE focused on how young people want or value SRE being provided and the attributes of educators they felt were needed for SRE to be effective.

#### Facilitators are Trustworthy, Credible, and Respectful

Overwhelmingly, young people reported that they needed adults and organisations providing SRE to be trustworthy and respectful. Trustworthy adults were those who were available to young people, who “*let you know that they [are] there for you, even when you wrong*” (Aparicio et al., 2021), who don’t “look down” on young people and who use the unequal power dynamic to support rather than control young people. Trustworthiness also related to credibility – with young people viewing “*doctors, school counsellors, and foster care staff as potential examples of trusted sources*” (Aparicio et al., 2021). Young people asserted that not all professionals were trusted or trustworthy and reported that to be trustworthy, professionals needed to be available, to know them well enough, to demonstrate that they would respect young people’s confidentiality and have spent time building rapport (Aparicio, Kachingwe, et al., 2019; Aparicio et al., 2021).

Respect was often coded in terms of “*respecting young people*,” “*respecting of their experience*,” “*respectful of young people’s pasts*.” Respectful facilitators were ones that were appreciative of young people, their perspectives and their agency and worked in a way that demonstrated that they considered young people as “equals” (Rew et al., 2002). Young people also felt that programmes were respectful when they acknowledged young people’s own expertise and experience and, in some cases, the fact that they were experts on issues such as relationships, sex and sexuality, and fostered opportunities for young people to learn from each other. Participants also stressed the importance of facilitators honouring young people’s pasts (including their maltreatment but also the ways that they had dealt with issues such as homelessness etc., [Aparicio, Kachingwe, et al., 2019; Aparicio, Shpiegel, et al., 2019; Aparicio et al., 2021]). Valued facilitators worked in a way that challenged preconceptions about “vulnerable” or “problem” groups. As Rew et al. (2002, p. 173) observed: *While homeless adolescents may look as if they are rebelling against established institutions, the youth in this study are clearly asking to be seen as persons worthy of society’s respect and of its best efforts to help them meet their needs and promote their sexual health.*

Finally, young people reported that to be trusted, adult educators needed to work non-judgmentally and in ways that challenged rather than perpetuated stigma: about maltreatment, about young people at the margins (e.g. those in care or homelessness services or who were younger parents); those that were using alcohol or other drugs, were selling sex, or engaging in risky sexual behaviours (Aparicio et al., 2021; Opara et al., 2022; Rew et al., 2002).

#### Trauma-Informed in Their Approach

As discussed above, young people reported experiencing maltreatment and have made some observations about how this had and continued to influence their lives. Workers in a few studies pointed to the fact that young people were often unaware of the link between their past maltreatment and their present situations, behaviours and attitudes towards sex and relationships. They suggested that central to work with these young people was helping them appreciate and overcome or find new ways of managing the ongoing impacts:

I think that [youth in care] may not fully understand how their past trauma is affecting their mental health and their views of sex and sexual activity, or why they do the things they do. So, I think it would be good if we could maybe delve more into trauma and behaviors as a result. (Aparicio et al., 2021, p. 1144)

In terms of the ways that SRE is facilitated, young people did make some reference to the need for educators to be patient and respectful of their pasts and ongoing trauma. Although none of the young people talked about “triggering” they did allude to the fact that sex education could be challenging for young people who had experienced maltreatment.

### What Attributes Do Young People Value About Programmes?

In addition to the attributes that young people valued in an educator, there were a few themes related to the nature of the programmes that were being provided or might better meet their needs.

#### Fostering Peer Sharing and Groupwork

In several groups, young people expressed the value of learning from and with others with lived experience – either of maltreatment, care, or sexual health issues. One group of young people valued “knowing everybody’s opinions about the topic” and believed that “it’s good to hear from other people [staff members and youth participants], because everybody has different advice” (Aparicio, Shpiegel, et al., 2019, p. 7).

In the Wahine Talk project (Aparicio, Kachingwe, et al., 2019), young people talked about the value of having a peer mentor who was available to provide guidance and encouragement both in terms of the project but also in relation to the sexual health information being offered.

In addition to appreciating the opportunities to learn from their peers, some young people stressed the value of learning from others with lived experience. Young people in Opara’s et al. (2022, p. 6) study believed that when receiving information about HIV, for example, that meeting adults who had contracted the virus and were living with it would help them better appreciate the condition while humanising and breaking down the stigma associated with it.

#### Outreach and Peer-Driven Projects

In three of the studies, young people expressed that they and their peers may have some hesitation about going to a particular location and advocated for outreach models: meeting and providing SRE to young people in the places where they lived or frequented.

Young people who had been through programmes also felt that they could play an active role in educating their peers and others in the community. Playing an active role and being part of a group experience was valued by young people in several projects. In Opara’s et al. (2022) paper, in particular, young people spoke about their interest in being able to use the knowledge that they had gathered and the skills that they built to support other young people who were in similar situations to themselves:

If you never gave us the space to talk about these things, we would have never realized that we have the power to do something about this. Even if we talk to just one of our friends, or share the knowledge with one person on the street, we are doing something right, someone can be saved (Opara et al., 2022, p. 6)

They believed that a peer-based outreach programmes would be valuable in that: “if you go out there, youth will listen to us because we have been there and can speak their language” (Opara et al., 2022, p. 6). Some young people were taken by the opportunities of playing a part and talked about how they might work with others to do so.

#### Complemented by Supports

In many of the studies, young people talked about the fact that they valued supports being provided alongside the sex and relationship programmes. In the case of Rew’s study, young people felt that they were more likely to utilise “safe sex” messages if they were provided condoms. Alternatively, some young people felt that sex education might be provided when young people were asking for condoms – at a sexual health clinic, for example: “*there should be a no-judgment zone where kids can ask for condoms . . . They can just make it available . . . and we can talk about sex*” (Opara et al., 2022).

Other young people talked about the value of being given food, and access to other forms of sexual health care such as sexually transmitted infection and pregnancy checks. These added “incentives” not only helped to encourage young people’s participation but acted to reinforce the things that were learned through programmes (Aparicio, Kachingwe, et al., 2019; Rew et al., 2002).

In addition, young people (and staff) were supportive of opportunities for their broader health needs to be met alongside SRE. As noted above, young people were mindful of the interplay between their drug use and problematic sexual behaviour and additional risk-taking (Lindroth, 2014). Others recognised that they may receive support when they fell pregnant, were seeking guidance about contraception (Aparicio, Kachingwe, et al., 2019; Aparicio, Shpiegel, et al., 2019; Aparicio et al., 2021; Rew et al., 2002) or had disclosed abuse or other safety concerns (McKibbin et al., 2017).

## Discussion and Implications for Practice

The recommendations made in the following sections might be used to complement the work of others who have demonstrated other areas of content and effective styles of facilitation that would be pertinent to work with these vulnerable groups (Goldfarb & Lieberman, 2021; Lameiras-Fernández et al., 2021; Pound et al., 2016; Unis & Sällström, 2020).

Our critical findings are included in Table 3, and the implications for policy and practice are included in Table 4.

**Table 3.** Summary of Critical Findings.

• Young survivors express a desire for comprehensive SRE covering topics such as adult-child and peer-to-peer behaviours, contraception, STI prevention, and reproductive health and services. • Some young people viewed sex in transactional terms, seeking intimacy for various needs like money, drugs, or security. However, there was a lack of emphasis on pleasure or love in these discussions. SRE might aim to reorient young people’s notions of healthy sexuality. • Tailored SRE programmes should acknowledge and address the nuanced experiences and needs of young survivors of maltreatment, including considerations of identity, aspirations, and relationship dynamics. • Given identified issues around masculinity, entitlement, and relationship expectations, a gendered approach to SRE is necessary to address gender roles and heteronormativity effectively. • SRE programmes, both universal and targeted, should adopt trauma-informed approaches, recognising the prevalence and impact of trauma on young people's experiences and behaviours.

*Note.* SRE = sexual and relationship education.

**Table 4.** Implications for Policy and Practice.

• Universal SRE needs to recognise that many young people will have experienced maltreatment, be responsive to their needs and provide pathways to trauma-informed services. • Targeted SRE programmes should be available at pivotal points in young people’s lives, such as before puberty, during disclosure of maltreatment, and during pregnancy, to provide timely support and education. • Targeted SRE programmes need to be trauma-informed, recognising and addressing young survivors’ trauma while providing opportunities for healing, growth, and reclaiming sexuality. • Educators should collaborate with young people to shape SRE programmes, involving them in content development, delivery (i.e. mentoring, peer support, and lived experience advisers), and implementation, to ensure relevance and effectiveness. • Targeted programmes should be complemented with additional supports addressing multiple layers of disadvantage faced by young people, such as homelessness and substance misuse, to provide holistic support.

*Note.* SRE = sexual and relationship education.

### Broad in Scope and Responsive to Young People’s Educational Needs

Young people in the various studies identified a series of content areas that they would like to see incorporated in SRE. It may be unrealistic to think that any one programme might cover the breadth of content suggested but during their adolescence, young people were keen to be provided with content that helps them understand: the nature, risks, and ways of identifying and responding to adult-child and peer-to-peer behaviours, including child abuse, inappropriate adult-youth relationships, sexual assault, and rape. Discussion on these topics might be facilitated in such a way that young people recognise not only the dynamics but also the ways in which these experiences can shape young people’s sense of self, their aspirations for relationships and sex and the ways that they interact with others. Helping them to create new expectations for themselves and providing them the means to recast sexuality to being something positive, pleasurable, and healthy (Fava & Bay-Cheng, 2013; Lameiras-Fernández et al., 2021; Panisch et al., 2020).

In terms of sexual health, content areas might cover learning related to safe sex, contraception, STI prevention and pregnancy – topics that are included in many existing SRE programmes (Pound et al., 2016). Such lessons might include not only how to negotiate the use of contraceptives in heterosexual and intimate relationships but also in scenarios of homosexual and brief sexual encounters and where there might be power imbalances or other mitigating factors (e.g. the selling of sex or under the influence of alcohol or other drugs). They should recognise that many young people have some knowledge about sex but also may hold false assumptions about things like contraceptives (e.g. that you can’t get pregnant the first time). Young people might not only be provided information about ways to protect themselves but, as importantly, how to access good sexual health information, how to access contraceptives (e.g. condoms and birth control), and to whom to turn if they want information or need to make informed decisions about pregnancy and reproductive health – content that is not always available in existing programmes (Lameiras-Fernández et al., 2021).

In terms of gender roles and heteronormativity, there was limited discussion among the groups about what lessons young people might want and need. However, given the attitudes and behaviours identified by some of the young people (e.g. around masculinity, male entitlement, and young men’s lack of responsibility in terms of birth control, etc.; and young women’s lower expectations about what types of relationships they might have), a gendered approach to SRE might be required (Lameiras-Fernández et al., 2021).

Young people sometimes flagged issues in their relationships: in forging them, in sustaining them, in managing negotiations about sex and non-sexual matters. Central to equipping them to have happy, healthy relationships is building young people’s aspirations in terms of what they can expect from their partners and within their partnership. Unfortunately, research elsewhere has reported that for young women, in particular, who have experienced abuse or been exposed to family or intimate personal violence, negative perceptions of self and low expectations of relationships are apparent and intrinsic in comments such as “girls like me don’t deserve better” and “what more could I expect?” (Moore & McArthur, 2022). Coupled with education about positive masculinities, young men might also be supported to challenge their own fears about being in relationships and provide them better ways of expressing themselves and managing crises (Colman & Widom, 2004).

In synthesising the papers, it became apparent that many of the included young people spoke about sex in transactional terms: using intimacy as a way of acquiring money, drugs, shelter, or security or to forge or sustain a relationship. This appeared to be influenced by young people’s past abuse and negative experiences of sex. Absent from discussions was a consideration of pleasure or of love, except in terms of the potential love a child might bring for isolated young women. Discussions about how sex can be healthy, positive, and fulfilling appears warranted (Allen, 2007; Hirst, 2013).

In exploring the growing body of studies that focus on what young people need from SRE, it is not difficult to see significant overlap in content areas identified as being necessary and valued. However, young people in the included studies felt that targeted SRE, at least, needs to understand the nuances of their experiences, to better identify and respond to the sexual health and relationship risks that they might encounter while tailoring content to their existing knowledge and level of experience.

Echoing findings from previous research with young people in school-based programmes (Pound et al., 2016), this review also stresses the importance of ensuring that adults close to young people (in terms of distance and connections) are also equipped to engage, build trust and provide adequate and appropriate sex and relationship information and guidance (Noorman et al., 2023). As has been argued by others, this support should be offered to parents, carers and trusted adults early (so that they can support children from an early age) and be underpinned by an understanding of child abuse and violence prevention (Russell et al., 2020). As SRE needs to be tailored to the unique needs of young people, parent education and workforce development strategies will need to appreciate the unique contexts of young people, their families and the services that surround them to ensure that they are responsive to learners’ needs and to build skills, knowledge, and confidence (Peter et al., 2015). Engaging parents, carers, and youth workers in complementary programs may also enable SRE providers to confidently provide positive sex and relationship lessons without concerns of resistance from those less-informed (e.g. parents [Russell et al., 2020]), particularly when discussing more sensitive topics (Jankovic et al., 2013; Noorman et al., 2023).

### Trauma and Maltreatment-Informed Universal and Targeted Programmes

#### Universal Programmes

Young people’s perspectives on their educational needs have implications for both universal and targeted services. Although all the papers reported on young people’s access to or preferences for supports to be provided through targeted programmes, many noted that they had received SRE in other settings (including school, community organisations, and care services) but reported that these were generally unhelpful and unresponsive to their needs. We recognise that not all universal programmes can meet the needs of all young people – however, findings from this synthesis would echo findings of previous research (Fava & Bay-Cheng, 2013; Panisch et al., 2020) and suggest that mainstream SRE programmes need to be aware of and provide openings for young people with multiple vulnerabilities to hear affirming messages and, preferably, to be referred on to targeted services. We also recognise that universal services are usually framed in terms of primary prevention (preventing abuse from occurring through programmes targeting the broad community) but, given the number of young people who have experienced maltreatment, do also have a secondary prevention role (supports for those who have experienced maltreatment or at greater risk of harm; Knack et al., 2019).

We would agree with others in asserting that universal programmes need to be, at the very least, trauma-informed. Historically, trauma-informed approaches have primarily aimed to “acknowledge the prevalence of trauma and the impact trauma has on learning and behaviour” (Pfitzner et al., 2022, p. 23). As such, trauma-informed approaches to SRE have aimed to: improve realisation and recognition among educators of trauma and its impacts on children and adolescents; improve educator responses to children and adolescents experiencing trauma; limit re-traumatisation of children and adolescents by increasing support and reducing punishments in education settings; and limit secondary/vicarious trauma among educators by increasing support and professional learning of educators concerning child and adolescent trauma” (Berger, 2019 in Pfitzner et al., 2022, p. 23).

However, we and others (Fava & Bay-Cheng, 2013; Panisch et al., 2020) would argue that trauma-informed work needs to be conceptualised more broadly and “need[s] to be inclusive and deliberate in including content that represents experiences of all youth in a way that is universally accessible, especially those who are more likely to experience trauma and stigmatisation” Panisch et al. (2020, p. 889). We recognise that it may be beyond the scope and capacity of many mainstream SRE programmes to provide young people with opportunities to heal and recover from their maltreatment but would argue that, at the very least, they should help young people find supports that enable them to do so.

#### Targeted Programmes

The papers reviewed describe and propose the need for targeted programmes for those who have experienced maltreatment as well as for those living in different contexts (e.g. homelessness services, care settings) and experiencing different types of challenges (e.g. young parenting, HSBs). These targeted programmes might be considered as part of a suite of secondary prevention offerings – that target and respond to the needs of those who have experienced maltreatment and/or at risk of experiencing harm (Knack et al., 2019).

As with generic SRE programmes (Hirst, 2013; Pound et al., 2016), targeted programmes need to be made available at pivotal points: preferably prior to puberty, when young people first disclose maltreatment, when these young people hit puberty and first start engaging in sexual relationships; and when they seek broader sexual health information and/or become pregnant.

Although the content proposed in the included papers does not differ significantly from those identified as vital in reviews of mainstream programmes (Pound et al., 2016), it would appear that there are significant nuances that need to be communicated when supporting young survivors with layered disadvantage.

Young people, authors and advocates also argue that these targeted programmes need to be more trauma-informed than universal services: taking steps to not only acknowledge young people’s trauma but to recognise and provide links to opportunities for young people to heal, grow, and recover from these experiences. In particular, these programmes need to acknowledge “past experiences of abuse, the promise of resilience, and young people’s right to positive sexualities” (Fava & Bay-Cheng, 2013, p. 1). As such, they need to provide young people with frameworks to reclaim their sexuality and to have aspirations for healthy sexual and intimate relationships (Panisch et al., 2020).

Drawing on the findings from this synthesis, we would argue that to be fully trauma-informed SRE also needs to: recognise the relationship between childhood maltreatment and a young person’s engagement in risk-taking and harmful behaviours; appreciate the ways that maltreatment can skew young people’s sense of identity, sexuality, and relationships and accommodate this in SRE design; and recognise the fact that young people who have previously experienced maltreatment are more likely to be exposed to further harm into the future. Like others (Fava & Bay-Cheng, 2013), we would argue that this work should not reinforce limiting notions of young survivors (and be blind to their resilience) but, instead, to be sensitive and guided by young people and their needs. Targeted programmes might be complemented with supports that help young people also deal with some of the multiple layers of disadvantage demonstrated in the papers included in this study: namely homelessness, alcohol and other drug misuse, etc.

### Participatory and Youth-Informed

In previous reviews of SRE in school settings, writers have recommended opportunities for young people to help shape content, delivery modes and locations and provide feedback on their strengths and weaknesses (Pound et al., 2016). This is particularly pertinent as educators (and, in fact researchers [Templeton et al., 2020]) often understand young people’s sexual health needs, and hold different views of quality and responsiveness to these needs, than young people themselves (Hirst, 2013). Young people in this review agreed and stressed the value of educators working alongside young people to not only shape programmes but also in playing a role in its implementation (Aparicio, Kachingwe, et al., 2019; Opara et al., 2022). Models of peer support, peer outreach, and peer mentoring (which have been appreciated and shown to be effective in other reviews) were all valued by young people.

## Limitations and Future Work

This paper drew together insights gleaned from young survivors about their experiences of and suggestions for improved SRE. When conceiving the project, we hoped to draw together the needs and experiences of a broad range of survivors – recognising that they come from all walks of life and would have a broad range of views that might inform future SRE work. However, the included studies engaged young people who, in addition to child maltreatment, had or were experiencing a range of challenges that may have amplified the impacts of their earlier abuse. We greatly appreciate their insights but need to acknowledge that they may not be representative of the needs or experiences of young people who may not be living on the margins. We also recognise that the existing literature does not include studies that focus on other groups of survivors and their intersectionality: including young survivors who are from First Nations communities, those with disability, and those from culturally or linguistically diverse backgrounds.

We also recognise that, although comprehensive, the included papers did not address contemporary issues related to sex and sexuality: namely, the risks of sexual exploitation and of unsafe risks online (e.g. sextortion and revenge porn). Recognising that young victim-survivors, particularly those engaged with child protection and homelessness systems (Moore et al., 2016), are vulnerable to these issues, future research might consider how SRE might also fill the gaps in such education.
